# Supplementary material for: Characteristics and trends of medical malpractice claims in Japan between 2006 and 2021
Source: PLoS One. 2023 Dec 18;18(12):e0296155. doi: 10.1371/journal.pone.0296155 (PMC10727369; doi:10.1371/journal.pone.0296155)
Supplement: Attachment — Submitted filename: Response to reviewers.docx [file pone.0296155.s002.docx]

Reviewer #1: All comments addressed. Well worded response to prior. Changes to other reviewer comments also made. Recommend acceptance for publication.

**Response**

Thank you for your thorough review of our paper. Your recommendation for acceptance for publication is greatly valued, and we are grateful for your support.

Reviewer #2: Thank you for allowing me to review this revision. The authors appear to have addressed my comments.

**Response**

We want to express our appreciation for your time and effort in reviewing the revised version of our paper. Your feedback and insights have been invaluable throughout this process, and we are pleased to hear that you found that the authors have addressed your comments to your satisfaction.

Reviewer #3: The authors replied to the reviewer's points properly except one point; the figures still have readability issues. For line graphs, you can put the shape of the line (e.g. dotted line or short-dashed line) as well as the color, or for box graphs, you can fill the box with a pattern.

**Response**

Thank you very much for your valuable feedback. In response to your comments, we have made changes to the line shapes for line graphs, such as dotted lines and short dashed lines. In addition, we have improved the visibility of box graphs by adding a distinctive pattern in the box. We are confident that the clarity of the charts can be communicated more effectively.

**Changes**

The box graphs in Figures 1 and 2 were changed to a pattern fill. The line graph in Figure 3 has been changed to a dotted line or a short-dashed line.　Figure 4 is also in black and white for consistency with the other graphs. Further, titles in Figures 2 and 3 have been deleted.

Fig1 (before correction) 　　Fig1 (revised)

Fig2 (before correction) 　　　Fig2 (revised)

Fig3 (before correction) 　　Fig3 (revised)

Fig4 (revised)

　　　　Fig4 (before correction)

Reviewer #4: This study contains valuable information. In past studies, the frequency of

medical judicial complaints has been investigated, but the trend of this frequency has been less studied. The following suggestions are recommended for　this research:

1- In the discussion section, it should be mentioned the preventive application of these judicial complaints in hospital places, etc.

**Response**

Thank you for providing valuable and significant feedback. As you have suggested, and in addition to implementing general measures to reduce medical litigation, we have incorporated the following insights from the discussion on patient engagement. The input from the reviewers has played a crucial role in enhancing our paper, and we are genuinely grateful for their contributions.

**Changes**

**Lines 341–349**

“To enhance patient safety and elevate healthcare quality through insights gained from medical malpractice claims, it will be increasingly crucial to foster improved communication between healthcare providers and patients in the future, with a dedicated emphasis on engaging patients actively [32]. Also, in order to decrease the occurrence of distressing medical errors that result in legal actions, healthcare organizations must grasp both patients' and healthcare professionals' viewpoints. Consequently, medical professionals can establish patient safety management protocols to delve into the causes of medical errors, implement corrective measures, and cultivate a culture of safety.”

2- Will the decrease in the frequency of medical lawsuits over time mean a decrease in the frequency of complaints and dissatisfaction of patients in hospitals and clinics, etc.? This topic will be addressed in the discussion section.

**Response**

Thank you for your insightful comments. The decrease in the frequency of medical lawsuits does not mean that the frequency of patient dissatisfaction with medical care is decreasing. However, the Medical ADR was established in 2007, and now there are 11 locations nationwide. This has allowed disputes to be handled outside of court, which may have reduced the number of cases leading to litigation. Therefore, the following modifications have been made.

**Changes**

**Lines　267–273S；**

Closed medical malpractice claims during the study period peaked in 2006 and have been fluctuating between 700–800 cases annually in recent years. However, the decrease in the number of medical lawsuits probably does not translate into a decrease in the frequency of patient complaints and dissatisfaction with medical care. Medical ADRs were established in 2007 and are now located in 11 locations nationwide; thus, the number of cases leading to lawsuits may have been suppressed as disputes and are now handled outside of court.
